# Insights into the Biotic Factors Shaping Ectomycorrhizal Associations

**DOI:** 10.3390/biology13121044

**Published:** 2024-12-13

**Authors:** Belkacem El Amrani

**Affiliations:** Lumbricidae, Improving Soil Productivity and Environment Unit (LAPSE), Higher Normal School (ENS), Mohammed V University in Rabat, Rabat P.O. Box 554, Morocco; elamranibelkacem@gmail.com; Tel.: +212-673248318

**Keywords:** ectomycorrhizal, symbioses, host plants, microbial interactions, plant and fungal diversity

## Abstract

Ectomycorrhizal (EM) associations are essential symbiotic relationships that contribute significantly to the health and functioning of forest ecosystems. This review examines the biotic factors that influence EM associations, focusing on plant and fungal diversity, host specificity, and microbial interactions. Firstly, the diversity of host plants and ectomycorrhizal fungi (EMF) is discussed, highlighting how the richness of these organisms affects the formation and success of EM symbioses. Next, host specificity is explored, with a focus on the complex relationships between EMF and their host plants. Microbial interactions are examined in depth, with sections on both positive and negative influences of bacteria and different fungal groups on EM formation. Overall, this review provides a comprehensive overview of the biotic factors that shape EM associations, offering insights into the mechanisms that underpin these critical ecological interactions and their broader implications for ecosystem management and restoration.

## Simple Summary

Ectomycorrhizal associations are important partnerships between certain types of fungi and tree roots that play a key role in keeping forests healthy. This review looks at the different living factors that affect these relationships. It starts by discussing the variety of trees and fungi, showing how the presence of different species can influence the success of these partnerships. Then, it explains how some fungi are more selective and form connections with specific trees, leading to unique, specialized relationships. This review also explores how other microbes, such as bacteria and other types of fungi, can either help or hinder these connections. This review provides a clearer understanding of what shapes these essential partnerships and why they matter for the health and recovery of forests.

## 1. Background

**Figure 1:**
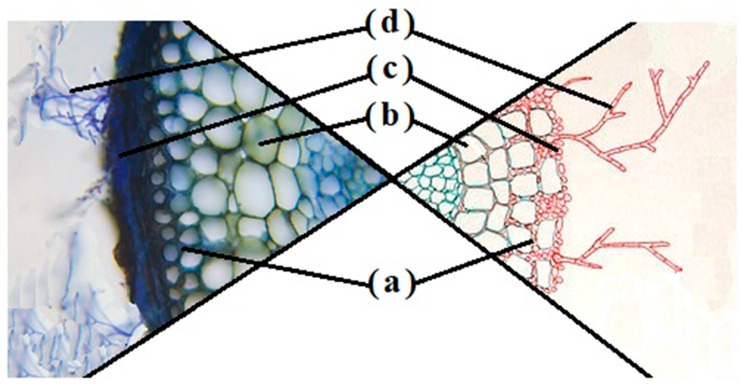
Cross-section and diagram of a cedar root colonized by an ectomycorrhizal fungus. (a) Hartig network, (b) plant cell, (c) fungal mantle, (d) extraradical hyphae.

Ectomycorrhizal (EM) associations are symbiotic relationships between fungi and the roots of host plants (Figure 1), which play a critical role in forest ecosystems. These symbiotic relationships are essential for nutrient cycling, plant health, and overall ecosystem function [1]. Ectomycorrhizal fungi (EMF) facilitate the uptake of nutrients, such as nitrogen and phosphorus, while benefiting from carbohydrates produced by their host plants [2]. The diversity and specificity of these associations are influenced by numerous factors, including plant diversity. High plant diversity can promote a greater range of fungal partners, enhancing overall ecosystem resilience and nutrient cycling [3]. More precisely, host specificity determines the selectivity of EMF for particular plant species, influencing the strength and efficiency of the symbiotic relationship. This specificity is influenced by environmental conditions, biochemical mechanisms, and evolutionary interactions [4]. Understanding the factors that govern host specificity is key to predicting how EM associations respond to changes in ecosystems. On the other hand, microbial interactions, including those with bacteria and other fungi, have significant effects on EM formation. Certain bacteria can facilitate the colonization of roots by EMF through mechanisms like the release of growth-promoting compounds, while others may hinder the process by competing for resources or producing inhibitory substances [5,6]. Similarly, interactions between different fungi can be either synergistic or antagonistic, affecting the stability and success of EM associations [7,8]. The study of EMF is crucial in the context of biodiversity conservation and sustainable land management. As ecosystems face increasing pressures from climate change, habitat loss, and other anthropogenic factors, the resilience and adaptability of EMF and their plant partners become vital areas of research.

This review aims to comprehensively explore the biotic factors that influence ectomycorrhizal associations, focusing on the roles of plant diversity, host specificity, and microbial interactions. By examining both positive and negative interactions between plants, fungi, and other microorganisms, this review seeks to elucidate the complex mechanisms that govern EM formation and their implications for ecosystem function.

## 2. Plant and Fungal Diversity

### Host Plant Diversity and EM Fungal Diversity

The identity of plant species plays a crucial role in shaping EMF community structure, as evidenced by numerous studies [9,10]. Dai et al. [11] highlighted that different plant species, through their unique root structures and biochemical profiles, create varying environments that influence fungal colonization patterns. For instance, variations in root exudates among plant species can attract and establish specific EMF, as shown by Lei et al. [12]. This selective attraction underscores the profound impact of host plant diversity on EM fungal diversity. In their study, Liu et al. [13] aggregated data on the global diversity of EMF, revealing that approximately 7750 fungal species form EM associations in the world, although the estimated species richness of EMF is likely between 20,000 and 25,000. These fungi, primarily from the Basidiomycota and Ascomycota phyla, belong to over 80 lineages and more than 250 genera. EMF are found in approximately 2% of vascular plants, especially in families like Pinaceae, Fagaceae, and Myrtaceae [13]. Brundrett and Tedersoo [3] emphasized that global host plant diversity, particularly at the family level, has a significant influence on the phylogenetic composition of EM fungal communities. Certain plant families, they argue, may promote a richer assemblage of these fungi, suggesting a strong co-evolutionary relationship. Moreover, plant communities with higher species diversity tend to support more diverse EM fungal assemblages, as demonstrated by Rudawska and Leski [14]. A meta-analysis also confirmed this relationship, showing that plant diversity explains 57.6% of the variation in EMF community composition across different forest ecosystems [15]. Within these communities, some plants act as ‘keystone’ species, with an outsized influence on fungal diversity, richness, and community structure [16]. As a result, host plant species are the first biotic factor significantly correlated with ectomycorrhizal species’ diversity and richness. It has been shown that replacing a native forest with an exotic tree species, although the replacement tree species may be colonized by the same fungus, generally reduces the distribution of native ectomycorrhizal fungi [17]. Moreover, the difference in the composition of host plants in the forest ecosystem gives rise to the differences in communities of EMF [18]. In addition to the direct effects of plant diversity, Eisenhauer et al. [19] found that root biomass and exudates influence soil bacterial and fungal biomass. This suggests that as plant diversity increases, so does the complexity of root interactions, which could enhance EMF diversity. This connection underscores the potential for plant diversity to create a more favorable environment for EMF through root-mediated processes.

The correlation between plant and fungal richness has been demonstrated in various studies. Saijo and Loo [20] reported a positive correlation between plant richness and fungal richness. This finding aligns with that of Zhang et al. [21], who demonstrated that the EM fungal community of *Quercus acutissima* exhibited a positive correlation with the richness of neighboring plants, particularly in mixed forests. In parallel, a higher diversity of both plants and EMF can lead to complementary resource use, where different species exploit distinct soil nutrients and niches, reducing competition and enhancing overall ecosystem productivity [19]. This diversity often results in functional redundancy, where multiple species perform similar ecological roles, thereby enhancing ecosystem stability by ensuring that essential functions, like nutrient cycling, continue even if some species are lost. The positive feedback loops between plant and fungal diversity are particularly important. This reciprocal relationship contributes to greater ecosystem resilience and productivity, allowing diverse plant–fungal associations to partition soil resources more finely and efficiently [22,23]. As a result, nutrient cycling and utilization within the ecosystem are optimized, leading to more sustainable and robust ecological systems. However, it is crucial to note that while plant diversity appears to benefit fungal diversity, the mechanisms behind this relationship require further exploration. For instance, the role of specific plant traits, such as nutrient use strategies, in influencing EMF diversity is not yet fully understood. Phillips et al. [24] highlighted that tree species typically associate with either arbuscular mycorrhizal fungi (AMF) or EMF, with each type employing distinct nutrient acquisition strategies, suggesting that nutrient acquisition strategies could shape fungal community structures. This insight opens avenues for future research to investigate how different nutrient strategies among host plants affect EMF diversity and function.

## 3. Host Specificity

### 3.1. Host Specificity and Ectomycorrhizal Associations

Host specificity in EMF is a crucial aspect of understanding the intricate relationships between fungi and their plant hosts. These relationships can involve both generalist and specialist fungi. Generalist EMF are capable of forming associations with a wide variety of host plant species, often across different families or even orders [25]. These fungi typically have a broad ecological tolerance and can thrive in diverse environments. Their ability to associate with multiple hosts makes them key players in maintaining the stability and resilience of EM networks, especially in ecosystems with high plant diversity. In contrast, specialist EMF exhibit a narrow host range, forming associations with only a few closely related plant species, or sometimes just a single species [25]. These fungi often have highly specific adaptations to their hosts, such as specialized hyphal structures or unique signaling pathways that facilitate root colonization. While specialists contribute to fine-tuned interactions within specific plant communities, they are more vulnerable to environmental changes or disruptions that affect their host plants. Moreover, recent studies have highlighted the varying levels of host specificity in the compatibility of EMF with different plant species, ranging from highly specialized associations (restricted to one or two plant species) to more generalist interactions [26]. However, despite the fact that the majority of ectomycorrhizal fungi are generalist [17], the structure of ectomycorrhizal fungal communities in forest ecosystems is strongly controlled by host plant preference and selectivity [27,28,29]. This compatibility is crucial for successful seedling colonization [30], ultimately shaping ecosystem resilience.

### 3.2. Host Specificity and Environmental Conditions

In a broader ecological context, Prieto-Rubio et al. [31] employed network analysis to investigate ectomycorrhizal fungal communities. Their findings revealed that the complexity of fungal interaction networks—defined by the number and strength of connections between fungal species and their plant hosts—significantly influences soil enzymatic activities. These enzymatic activities, in turn, mediate nutrient cycling and soil health, linking host specificity in EMF to broader microbial network dynamics. This suggests that host specificity in EMF is not an isolated trait but is shaped by and contributes to the functional complexity of microbial networks. While genetic factors play a significant role in determining host specificity, environmental conditions can also influence the extent and nature of ectomycorrhizal associations [4]. Soil characteristics, climate, and biotic interactions all contribute to the availability and distribution of both EMF and their host plants [32]. In nutrient-poor soils, for example, plants may rely more heavily on specific EMF that are particularly efficient at nutrient acquisition. This can lead to a tighter specificity between the plant and the fungus. Conversely, in nutrient-rich environments, plants might form associations with a broader range of EMF, reducing the degree of host specificity. Additionally, environmental stressors such as drought, salinity, or pollution can impact host specificity by altering the physiological state of the host plants or the availability of compatible EMF [4]. In some cases, plants under stress may shift their EM associations toward more stress-tolerant fungi, even if those fungi are not their typical partners in less challenging conditions [33,34]. For instance, studies on Pinus species indicate that under prolonged drought, these trees often form associations with more drought-tolerant fungal species, such as *Geopora pinyonesis*. These fungi enhance the tree’s water and nutrient uptake capacity in arid environments, replacing less drought-adapted EMF partners [35]. Similarly, a study on *Quercus mongolica* found a shift toward salinity-tolerant EMF such as *Gomphidius viscidus*. These associations provide critical survival benefits in salt-stressed soils, even at the cost of reduced reliance on typical fungal partners [36]. Pollution, particularly from heavy metals or industrial effluents, has been shown to disrupt host specificity. For example, in areas contaminated by cadmium and lead, plants such as *Betula* spp. establish relationships with EMF like *Suillus luteus*, which are adept at heavy metal sequestration and detoxification [37]. This suggests that the ecological flexibility and activity of ectomycorrhizal fungi depends on their genotypes, environmental factors, host plant genotypes, and the interactions between all these factors [32].

### 3.3. Biochemical Mechanisms of Host Specificity

Host specificity in plant–mycorrhiza interactions is a complex phenomenon influenced by various biochemical mechanisms. In the context of mutualistic interactions, such as those with mycorrhizal fungi, plants actively communicate with their symbiotic partners through the secretion of root exudates. These exudates, which include phenolics, amino acids, terpenoids, and sugars, play a critical role in shaping the rhizosphere microbiome and promoting beneficial symbioses. For instance, flavonoids like strigolactones have been shown to enhance AMF colonization, facilitating nutrient exchange between the plant and fungus [38,39]. Given their role in promoting AMF colonization, strigolactones may also play a role in enhancing EMF colonization, although this potential requires further investigation. For example, the suppression of flavonoid and phenylpropanoid secretion has been observed to inhibit the colonization of certain mycorrhizal fungi, highlighting the selective nature of these interactions [40]. On the other hand, Jiarui and Haisheng [41] demonstrated that the formation and maintenance of EM symbioses rely on complex molecular signaling processes between fungi and host plants. Signaling molecules, such as lipophilic chitin oligosaccharides (LCOs), play a crucial role in the early stages of communication, enabling plants to identify potential symbiotic partners. Effector proteins, including MiSSP7 from *Laccaria bicolor*, are secreted to suppress host immune responses and promote symbiosis by targeting specific host regulatory pathways. Additionally, plant cell wall-degrading enzymes, such as GH5 and GH28, contribute to fungal penetration into root tissues and the formation of Hartig nets, which are essential structures for nutrient exchange. This selective recruitment of mycorrhizal partners by the host plant underscores the intricate balance between defense and symbiosis, where plants must finely tune their biochemical pathways to support beneficial microbes while deterring potential pathogens.

## 4. Microbial Interactions

### 4.1. Beneficial Effects of Bacteria on EM Formation

As one of the most abundant and diverse groups of soil microbiome, bacteria engage in various interactions with EMF, influencing their behavior and ecological roles.

**Figure 2:**
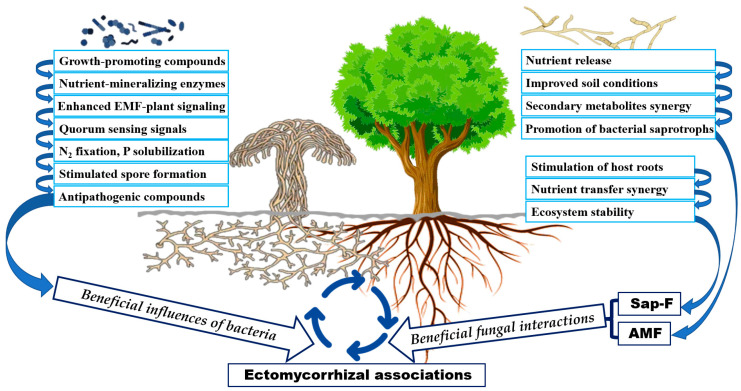
Illustration of the beneficial effects of bacteria and fungi on EM formation. EMF: ectomycorrhizal fungi; Sap-F: saprotrophic fungi; AMF: arbuscular mycorrhizal fungi.

The establishment of EM associations can be positively influenced by specific bacterial communities that facilitate the signaling processes essential for symbiosis (Figure 2). Certain bacteria, specifically mycorrhiza helper bacteria (MHB), have been identified as facilitators of EM fungal growth. Species such as Pseudomonas and Bacillus are known to enhance the EM associations through the production of growth-promoting substances like indole-3-acetic acid and gibberellins. These compounds not only stimulate fungal growth but also improve the overall health of the plant-fungal symbiosis [5]. MHB can be classified into two main functional groups: those that impact the functions of an already established EMF symbiosis and those that stimulate the initial establishment of fungal symbionts on host plants. These bacteria are typically found in the hyphosphere, mycorrhizosphere, and sporocarps (the fruiting bodies of fungi), particularly those formed by ectomycorrhizal fungi near root systems [42,43]. Examples include sporocarps of genera such as Amanita, Boletus, and Russula, which are closely associated with the roots of their host plants. MHB are predominantly found among Gram-negative Proteobacteria, such as Agrobacterium, Azospirillum, Burkholderia, and Pseudomonas, and among Gram-positive Actinobacteria and Firmicutes, including genera like Streptomyces, Bacillus, and Paenibacillus. These bacteria are known for their diverse metabolic activities, such as nitrogen fixation, phosphate solubilization, and phytohormone production, which contribute to enhanced mycorrhizal colonization and plant growth [44,45]. Frey-Klett et al. [46] demonstrated that MHB play a critical role in facilitating the establishment and functioning of mycorrhizal symbioses, enhancing fungal colonization efficiency and potentially boosting spore production. Additionally, MHB can influence root exudation patterns, which in turn alter the chemical signals that attract mycorrhizal fungi to the root surface. This modification of root exudates improves fungal attachment and colonization, particularly during the early stages of symbiosis, facilitating a more effective establishment of the mycorrhizal relationship [47]. Moreover, some MHB produce antimicrobial compounds that protect both the mycorrhizal fungi and plant roots from soil-borne pathogens. This protective role ensures the stability and functionality of the mycorrhizal association by reducing competition between EMF and pathogenic microbes, thereby providing a safer environment for the plant–fungus symbiotic relationship to thrive [47,48]. MHB also engage in complex microbial interactions, such as quorum sensing (QS), which involves the secretion of chemical signals that regulate microbial interactions and gene expression. Through QS, MHB can communicate with other microbes in the rhizosphere, coordinating their activities to enhance the establishment and functioning of mycorrhizal symbiosis [49,50]. In some cases, MHB are involved in direct physical interaction with mycorrhizal fungi, such as in producing specific sugars or enzymes that stimulate spore germination and fungal growth. Schrey et al. [51] demonstrated that the mycorrhiza helper bacterium (*Streptomyces* sp. AcH 505) plays a crucial role in facilitating ectomycorrhizal associations by promoting fungal growth and influencing the expression of specific genes in the ectomycorrhizal fungi *Amanita muscaria* and *Suillus bovinus*. This involves upregulating genes linked to symbiosis establishment and fungal development, while downregulating others associated with stress responses or competition.

Bacterial biofilms present another layer of complexity in microbial interactions with EMF. Biofilms can serve as protective environments for beneficial bacteria, enhancing their survival and functionality in the soil [52]. However, biofilms can also contribute to resource competition among microbial communities [53]. Given that EMF are integral members of these communities, this competition may also affect the success of EM association. Röttjers and Faust [54] discussed how microbial networks can influence community dynamics, suggesting that the interconnectedness of different microbial species can have profound implications for EM fungi’s ability to establish partnerships with plant roots. Moreover, the utilization of microbial inoculants in agriculture has garnered interest as a potential strategy to enhance EM associations. Santos et al. [55] reviewed the historical context and current applications of beneficial bacteria in agriculture. They suggested that targeted microbial inoculation has the potential to enhance EM formation and function in various agricultural settings.

### 4.2. Negative Effects of Bacteria on Mycorrhizal Formation

**Figure 3:**
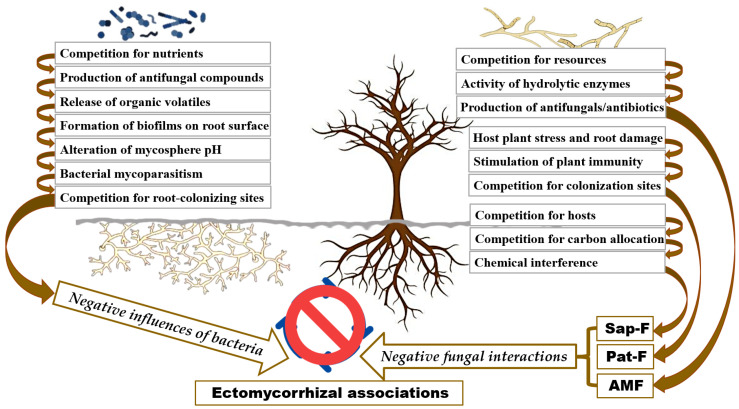
Illustration of the negative effects of bacteria and fungi on EM formation. Sap-F: saprotrophic fungi; Pat-F: pathogenic fungi; AMF: arbuscular mycorrhizal fungi.

While some bacteria support mycorrhizal formation, others can inhibit or disrupt these associations through various mechanisms (Figure 3). One of the primary mechanisms through which bacteria negatively affect mycorrhizal formation is by competing with EMF for essential nutrients. This competition not only affects the growth of EMF but may also lead to reduced plant vigor and health. EMF rely on carbon from their host plants, which is supplied in the form of simple sugars like glucose. Bacteria in the rhizosphere can also utilize these sugars, leading to direct competition with EMF. For example, studies have shown that Pseudomonas species, which are common in the rhizosphere, can efficiently metabolize plant-derived carbon, such as sugars, amino acids, and organic acids [56], potentially limiting the carbon available to EMF and thus inhibiting their growth and ability to form mycorrhizal associations, especially in carbon-limited environments. Ectomycorrhizal fungi have developed a variety of extracellular enzymes, such as cellulases and proteases, which are instrumental in breaking down organic matter and liberating nitrogen to access organic nitrogen sources in the soil [2,57]. However, soil bacteria can also mineralize organic nitrogen into forms that are easily accessible to plants and other microbes. Some bacteria can outcompete EMF for nitrogen by rapidly metabolizing these compounds, potentially reducing the nitrogen available to the fungi [6]. Hartmann et al. [58] demonstrated that roots and hyphae significantly reduce respiration rates, likely due to competition for soil nitrogen with other microbial communities. However, phosphorus is another key nutrient that both bacteria and EMF compete for. EMF produce phosphatases to liberate inorganic phosphate from organic compounds, but phosphate-solubilizing bacteria (PSB) can also mobilize phosphorus through the secretion of organic acids and enzymes [59]. Through this competition between PSB and EMF, PSB can outcompete EMF [60] by rapidly mineralizing these nutrients, making them less available to the fungi or/and can affect phosphorus availability to the host plant, thereby influencing plant growth. This competition can be particularly detrimental in nutrient-poor soils, where the availability of phosphorus is already limited.

Bacteria can inhibit EMF by producing antimicrobial compounds that directly affect fungal growth, such as antibiotics, siderophores, and lytic enzymes. For instance, Pseudomonas fluorescens is known to produce phenazine-1-carboxylic acid (PCA) [61], an antibiotic that inhibits the growth of large hyphal networks and often acts as an antifungal agent. This compound could potentially disrupt EM formation. These antagonistic behaviors are not limited to non-volatile compounds; volatile organic compounds (VOCs) produced by certain bacteria can also inhibit the growth and activity of EMF, as demonstrated by Effmert et al. [62]. Additionally, certain bacteria produce siderophores, which are molecules that bind and sequester iron from the environment [63]. Iron is a critical micronutrient for both bacteria and fungi, and the competition for iron can negatively impact EMF. Understanding these interactions and the specific mechanisms involved is crucial for developing strategies to manage soil ecosystems and enhance symbiotic development.

Bacteria can also physically disrupt the hyphal structures of EMF, which are crucial for root colonization and nutrient exchange. One mechanism of disruption involves the production of enzymes by chitinolytic bacteria, which degrade the chitin in fungal cell walls, leading to hyphal lysis and compromising the integrity of EMF. Research has shown that various bacterial taxa, including those from the genera Bacillus and Serratia, exhibit strong chitinolytic activity [64]. The ability of these bacteria to produce chitinases enhances their effectiveness in degrading fungal cell walls, as evidenced by the findings of Zhang et al. [65], who noted the presence of multiple chitinase genes in certain bacterial species. This enzymatic activity can significantly reduce the fungi’s ability to establish stable mycorrhizal associations. Additionally, certain bacteria, such as those belonging to the genus Bacillus, can form biofilms on the root surface, creating a physical barrier that prevents EMF from accessing the roots. The protective role of Bacillus biofilms was further demonstrated by Zhu et al. [66], who showed that *Bacillus pumilus* HR10 effectively colonizes the roots of pine seedlings through the formation of its biofilms.

Bacterial activity in the soil can alter its chemical environment in ways that are detrimental to EMF. One significant mechanism involves pH modulation, where certain bacteria produce organic acids as metabolic byproducts, leading to a decrease in soil pH [59]. The pH modification of the rhizosphere, such as through the production of gluconic acid by Pseudomonas species [67], can negatively affect EMF. Although certain EMF, such as *Xerocomus ferrugineus*, thrive in acidic soils with pH levels ranging from 3.5 to 6.5, many EMF prefer pH values around neutral. For instance, *Amanita rubescens* and *Suillus luteus* grow best at higher pH levels (6.5–8.5) [68]. Similarly, *Rhizopogon luteolus* and *Suillus luteus* perform optimally at pH 5.8–6.8 [69]. Therefore, lower pH levels can harm these groups of fungi through several mechanisms, such as disrupting cellular processes and nutrient uptake [70]. Acidification may alter the availability of essential nutrients; for example, while phosphorus becomes more soluble in acidic conditions, other nutrients such as calcium and magnesium may become less available [71], negatively impacting fungal growth. In addition to pH changes, bacterial metabolism, such as that of Pseudomonas, can generate toxic metabolites like hydrogen cyanide (HCN) and ammonia [72], that are harmful to EMF, thereby impairing their ability to establish and maintain mycorrhizal associations.

Direct antagonistic interactions between bacteria and EMF play a significant role in inhibiting mycorrhizal formation and affecting plant health. One such interaction is bacterial parasitism, where certain bacteria exploit EMF for nutrients while causing structural damage. For example, mycoparasitic bacteria like Lysobacter species produce extracellular lytic enzymes that degrade fungal hyphae, leading to a decline in fungal biomass [73]. This degradation could potentially target EMF hyphae as well, thereby hampering the formation of ectomycorrhizal associations. Additionally, bacteria can inhibit EMF by competing for root colonization sites, effectively occupying the same ecological niche and preventing symbiotic relationships from establishing. Pseudomonas species exemplify this behavior by outcompeting EMF for attachment sites on plant roots [74], thereby disrupting the formation of beneficial mycorrhizal associations. Moreover, Levy et al. [75] highlighted the genomic adaptations of bacteria that allow them to efficiently colonize plant roots. Their results suggest that these adaptations may also include mechanisms to outcompete fungi for space.

### 4.3. Beneficial Interactions Between Fungi in EM Formation

Recent research highlights the complex interactions influencing EM formation, with notable contributions from different fungal groups, such as saprotrophic fungi and AMF (Figure 2). While mycorrhizal–saprotrophic interactions can be either inhibitory or stimulatory depending on the context, these relationships are vital for understanding the broader ecosystem processes that govern nutrient cycling and plant–soil interactions [76,77].

Saprotrophic fungi are essential decomposers in forest ecosystems, breaking down complex organic matter such as leaf litter, wood, and other plant debris. This decomposition process releases a range of nutrients, including nitrogen, phosphorus, and carbon, into the soil, which can be readily utilized by EMF [78]. For example, species like Trichoderma and Penicillium have been shown to decompose organic phosphorus compounds, making phosphorus more available to EMF, which typically have limited access to this nutrient in organic form [56]. The released nutrients are then absorbed by EMF and transferred to their host plants, enhancing plant growth and health [8]. Moreover, the relationship between saprotrophic and EMF can be synergistic. Saprotrophic fungi not only provide essential nutrients but also alter the soil environment in ways that favor EM colonization. For instance, the breakdown of organic matter by saprotrophic fungi can lower soil pH, a condition that often promotes EM formation, but not below pH of 5, which is the minimum pH typically needed by most mycorrhizal fungi for effective symbiotic relationships [79]. Additionally, some saprotrophic fungi may produce secondary metabolites that stimulate EM fungal growth or suppress potential competitors, further facilitating EM associations [80]. Another form of positive interaction is the spatial niche differentiation between EMF and saprotrophic fungi. In boreal forests, EMF and saprotrophs may coexist by occupying distinct spatial niches, enabling both fungal groups to thrive without direct competition [81]. Furthermore, saprotrophic fungi may promote the activity of bacterial saprotrophs, which are capable of decomposing complex carbon substrates, including fungal and bacterial necromass [82,83]. This synergistic relationship enhances overall decomposition processes and contributes to the nutrient dynamics observed in EM and arbuscular mycorrhizal (AM) ecosystems. The activity of both saprotrophic bacteria and fungi may be necessary for the complete decomposition of plant and microbial biomass, highlighting the importance of these positive interactions in forest soil ecosystems [83]. These positive interactions highlight the intricate interdependence between different fungal groups, which is crucial for the successful formation and persistence of EM associations in soil ecosystems.

Different types of mycorrhizal associations, such as AMF and EMF, often coexist within the same ecosystem and even on individual trees [84,85]. While these mycorrhizal types were traditionally viewed as competitors, recent studies suggest that they can facilitate each other’s establishment and function [56]. These positive interactions enhance plant–soil functioning and contribute to greater multifunctionality within soil communities.

In various ecosystems, particularly mixed-forests, AMF, typically associated with herbaceous plants, and EMF, primarily found in woody plants, can interact and facilitate each other within shared mycorrhizal networks. These interactions facilitate the transfer of nutrients and signals between different plant species, enhancing EM colonization in mixed-species forests. For instance, AMF may initially colonize herbaceous plants, improving soil conditions and nutrient availability [86,87], which subsequently benefits EMF associated with nearby trees. This, in turn, enhances the establishment and survival of EM trees. This relationship promotes diverse plant communities and enhances overall ecosystem resilience [88]. Therefore, the presence of AMF directly influences EM associations by ameliorating the soil microbiome and nutrient dynamics. For example, AMF enhance phosphorus availability, which supports EM formation on adjacent trees [56]. Additionally, mycorrhizal networks that include both AMF and EMF contribute to ecosystem stability by supporting diverse plant communities [89], indirectly promoting EM establishment and persistence. The synergistic interactions between AMF and EMF extend beyond their influence on plant communities to significantly impact plant–soil functioning. When AMF and EMF grow together on the same root system, they can exert a synergistic effect on plant–soil interactions, enhancing nutrient uptake, soil structure, and overall plant health. This synergy is particularly evident under extreme environmental fluctuations, such as varying soil moisture, nutrient availability, and temperature. EMF may dominate under mesic conditions, while AMF prevail under extreme soil moisture conditions, high temperatures, and elevated nutrient availability. This complementary colonization optimizes nutrient uptake and enhances plant stress resilience [85]. These two symbioses utilize different nutrient acquisition strategies that can complement each other. This functional diversity enhances ecosystem nutrient cycling, enabling the efficient management of resources and maintaining productivity across varying environmental conditions.

The beneficial interactions between fungi involved in EM formation are of critical ecological and practical significance. Understanding these interactions offers valuable insights for forest management and restoration, especially in environments with limited nutrient availability. Indeed, integrating saprotrophic fungi into soil amendments or restoration efforts can accelerate nutrient cycling, thereby supporting EM formation in newly planted forests. Additionally, creating conditions that allow different mycorrhizal types to coexist may boost overall forest health and productivity. This approach is especially beneficial in degraded lands where EMF might face challenges in establishing robust colonization and functioning without the synergistic support of other fungal partners. Nevertheless, more research is needed to fully understand the complex interactions between different fungal types and their impact on EM formation. Furthermore, exploring the molecular mechanisms behind these fungal interactions could pave the way for new biotechnological innovations in forestry and agriculture [56]. Ultimately, the positive interactions between fungi—particularly the contributions of saprotrophic fungi to nutrient cycling and the facilitation between different mycorrhizal types—play a critical role in EM formation.

### 4.4. Negative Fungal Interactions on EM Formation

Saprotrophic fungi, which rely on decomposing organic matter for their sustenance, can also negatively impact mycorrhizal fungi through various competitive interactions (Figure 3). In particular, saprotrophic fungi often compete with EMF for essential nutrients and carbon sources, especially in nutrient-poor environments [90]. This competition can hinder the establishment and growth of EM associations by reducing the availability of resources that are critical for the formation and maintenance of mycorrhizal networks. The outcome of this competition is influenced by factors such as the relative abundance of saprotrophic and EMF, as well as the availability of organic matter and nutrients in the soil. In nutrient-rich environments, saprotrophic fungi dominate in their ability to degrade organic matter due to the higher abundance of carbohydrate-active enzymes (CAZymes) compared to EMF [91]. This enzymatic advantage allows saprotrophs to access and transform complex organic nutrients more efficiently. Additionally, some saprotrophic fungi produce secondary metabolites or antimicrobial compounds that can inhibit the growth or spore germination of mycorrhizal fungi, thereby reducing their colonization potential [92]. Moreover, the presence of saprotrophic fungi influences ecosystem processes beyond direct competition for resources. Research by Rousk and Bååth [93] highlighted that saprotrophic fungi interact with soil bacteria. This interaction introduces additional complexity to their ecological relationships, particularly with mycorrhizal fungi. These interactions can disrupt the mutualistic relationship between plants and mycorrhizal fungi, potentially leading to decreased plant nutrient uptake and overall ecosystem productivity.

Pathogenic fungi can have detrimental effects on mycorrhizal fungi, disrupting the symbiotic relationships between these fungi and their host plants through direct antagonism and competition (Figure 3). These pathogenic fungi may compete with EMF for root colonization sites. This type of competition has been demonstrated in *Fusarium oxysporum*, where the pathogenic and nonpathogenic (saprophytic) strains, representing two different trophic modes, compete for infection sites on tomato roots [94]. Such interactions can further limit the extent of mycorrhizal associations, reducing their overall effectiveness. This competition can be particularly intense in environments where pathogenic fungi are abundant, as they may occupy critical root zones that would otherwise be colonized by mycorrhizal fungi. Pathogenic fungi are known to produce toxins, enzymes, or other compounds that degrade fungal cell walls; although direct evidence for EMF degradation is limited, this hypothesis aligns with established mechanisms of fungal competition and warrants further investigation. The negative effects of pathogenic fungi are further compounded by the immune responses they trigger in plants. When a plant is infected by pathogenic fungi, it often produces defensive compounds to combat the infection. These compounds include reactive oxygen species (ROS), antimicrobial enzymes, and various secondary metabolites like phytoalexins [95]. These compounds help restrict the growth and spread of the pathogen. However, these compounds can also inadvertently inhibit the growth of beneficial mycorrhizal fungi, reducing their ability to colonize roots and form effective symbiotic associations. This collateral damage weakens the overall mycorrhizal network, diminishing its ecological benefits, such as enhanced nutrient cycling and improved plant health. Pathogenic fungi may also disrupt the signaling and recognition processes that are crucial for the establishment of ectomycorrhizal associations [96]. The presence of pathogenic fungi can weaken the plant host [97], further compromising its ability to support ectomycorrhizal fungi and thereby reducing the overall health and resilience of the mycorrhizal symbiosis.

Negative interactions between different mycorrhizal fungi, particularly between AMF and EMF, can significantly influence the dynamics of plant–fungal symbioses (Figure 3). These interactions are often characterized by competition for root colonization sites and access to essential resources, such as carbon from host plants and nutrients from the soil. This competition can lead to reduced colonization rates and the effectiveness of both AMF and EMF when they coexist in the same environment [98]. This competition can limit the availability of root niches for EMF, thereby reducing their establishment and proliferation. Furthermore, AMF can influence how the host plant allocates resources, potentially limiting the availability of nutrients and carbohydrates for EMF. This resource competition can result in suboptimal plant growth and a decline in the establishment of EM associations, potentially shifting the overall composition of the mycorrhizal community. The antagonistic interactions between AMF and EMF can also be mediated by allelopathic compounds or other inhibitory substances produced by one type of mycorrhizal fungi to suppress the growth or spore germination of competing species [98]. The outcome of these competitive interactions is influenced by various factors, including the relative abundance and interactions of different mycorrhizal types, soil resource availability, and the specific adaptations of the fungal species involved. For example, in nutrient-poor environments, EMF may have a competitive advantage due to their ability to efficiently mobilize and scavenge nutrients from organic matter, giving them an edge over AMF [98,99]. However, in other contexts, AMF might dominate, leading to different impacts on EM formation and overall mycorrhizal network functioning. Therefore, understanding the nuanced interplay between coexisting mycorrhizal fungi is crucial for elucidating the complex factors that shape the formation and dynamics of EM associations, as well as the broader ecological roles that mycorrhizal fungi play in ecosystems.

## 5. Conclusions

The intricate interplay between plant and fungal diversity, host specificity, and microbial interactions underpins the complexity of EM associations. This study highlights the importance of plant and fungal diversity in shaping EM fungal communities, emphasizing that host specificity is not only a driver of these associations but also a critical determinant of ecosystem function. The interactions between EMF and bacteria, both positive and negative, play significant roles in modulating the success and efficiency of mycorrhizal symbiosis. Positive bacterial influences, such as the promotion of fungal growth and symbiosis, are counterbalanced by negative interactions that can disrupt these associations through competition, antimicrobial production, and direct antagonism. Additionally, the role of fungal–fungal interactions, whether synergistic or antagonistic, further adds to the complexity of EM formation and its ecological implications. Despite the growing body of research on biotic interactions influencing EM associations, future research should focus on understanding the biochemical and molecular mechanisms of host specificity in EM associations, exploring how environmental changes impact these interactions, and investigating the role of microbial interactions in soil health. Additionally, studies should examine the facilitation between different mycorrhizal types to optimize plant growth in challenging environments and assess the ecosystem-level impacts of EMF, particularly their roles in carbon sequestration, nutrient cycling, and biodiversity conservation, to inform sustainable land management and conservation strategies.

## Data Availability

Data is contained within the article.

## References

[1] El Amrani B. (2022). Effects of Soil Biotic and Abiotic Properties on the Growth and Mycorrhization of Cedars, Cedrus Atlantica Manetti. Bois Forêts Trop..

[2] Tunlid A., Floudas D., Op De Beeck M., Wang T., Persson P. (2022). Decomposition of Soil Organic Matter by Ectomycorrhizal Fungi: Mechanisms and Consequences for Organic Nitrogen Uptake and Soil Carbon Stabilization. Front. For. Glob. Chang..

[3] Brundrett M.C., Tedersoo L. (2018). Evolutionary History of Mycorrhizal Symbioses and Global Host Plant Diversity. New Phytol..

[4] Van Der Linde S., Suz L.M., Orme C.D.L., Cox F., Andreae H., Asi E., Atkinson B., Benham S., Carroll C., Cools N. (2018). Environment and Host as Large-Scale Controls of Ectomycorrhizal Fungi. Nature.

[5] Raaijmakers J.M., De Bruijn I., Nybroe O., Ongena M. (2010). Natural Functions of Lipopeptides from Bacillus and Pseudomonas: More than Surfactants and Antibiotics. FEMS Microbiol. Rev..

[6] Tatsumi C., Taniguchi T., Du S., Yamanaka N., Tateno R. (2020). Soil Nitrogen Cycling Is Determined by the Competition between Mycorrhiza and Ammonia-oxidizing Prokaryotes. Ecology.

[7] Boddy L., Hiscox J. (2016). Fungal Ecology: Principles and Mechanisms of Colonization and Competition by Saprotrophic Fungi. Microbiol. Spectr..

[8] Lindahl B.D., Tunlid A. (2015). Ectomycorrhizal Fungi—Potential Organic Matter Decomposers, yet Not Saprotrophs. New Phytol..

[9] Yang T., Adams J.M., Shi Y., He J., Jing X., Chen L., Tedersoo L., Chu H. (2017). Soil Fungal Diversity in Natural Grasslands of the Tibetan Plateau: Associations with Plant Diversity and Productivity. New Phytol..

[10] Santolamazza-Carbone S. (2021). Ectomycorrhizal Fungal Community Structure in a Young Orchard of Grafted and Ungrafted Hybrid Chestnut Saplings. Mycorrhiza.

[11] Dai D.-Q., Suwannarach N., Bamunuarachchige T.C., Karunarathna S.C. (2023). Editorial: Plant-Fungal Interactions. Front. Microbiol..

[12] Lei X., Shen Y., Zhao J., Huang J., Wang H., Yu Y., Xiao C. (2023). Root Exudates Mediate the Processes of Soil Organic Carbon Input and Efflux. Plants.

[13] Liu Y., Li X., Kou Y. (2020). Ectomycorrhizal Fungi: Participation in Nutrient Turnover and Community Assembly Pattern in Forest Ecosystems. Forests.

[14] Rudawska M., Leski T. (2021). Ectomycorrhizal Fungal Assemblages of Nursery-Grown Scots Pine Are Influenced by Age of the Seedlings. Forests.

[15] Yang N., Hua J., Zhang J., Liu D., Bhople P., Li X., Zhang Y., Ruan H., Xing W., Mao L. (2022). Soil Nutrients and Plant Diversity Affect Ectomycorrhizal Fungal Community Structure and Functional Traits across Three Subalpine Coniferous Forests. Front. Microbiol..

[16] Fei S., Kivlin S.N., Domke G.M., Jo I., LaRue E.A., Philips R.P. (2022). Coupling of Plant and Mycorrhizal Fungal Diversity: Its Occurrence, Relevance, and Possible Implications under Global Change. New Phytol..

[17] O’Hanlon R., Harrington T.J. (2012). Similar Taxonomic Richness but Different Communities of Ectomycorrhizas in Native Forests and Non-Native Plantation Forests. Mycorrhiza.

[18] Chai D.-D., Guo S.-J., Sun X.-B., Qin T.-T. (2013). The Major Factors Affecting Ectomycorrhizal Fungi Diversity in the Forest Ecosystem. Adv. J. Food Sci. Technol..

[19] Eisenhauer N., Lanoue A., Strecker T., Scheu S., Steinauer K., Thakur M.P., Mommer L. (2017). Root Biomass and Exudates Link Plant Diversity with Soil Bacterial and Fungal Biomass. Sci. Rep..

[20] Saijo Y., Loo E.P. (2020). Plant Immunity in Signal Integration between Biotic and Abiotic Stress Responses. New Phytol..

[21] Zhang W., Xue W., Liu J., Zhu H., Zhao Z. (2024). Molecular Diversity of Ectomycorrhizal Fungi in Relation to the Diversity of Neighboring Plant Species. Microorganisms.

[22] Molina R., Horton T.R., Horton T.R. (2015). Mycorrhiza Specificity: Its Role in the Development and Function of Common Mycelial Networks. Mycorrhizal Networks.

[23] Heilmann-Clausen J., Maruyama P.K., Bruun H.H., Dimitrov D., Læssøe T., Frøslev T.G., Dalsgaard B. (2016). Citizen Science Data Reveal Ecological, Historical and Evolutionary Factors Shaping Interactions between Woody Hosts and Wood-inhabiting Fungi. New Phytol..

[24] Phillips R.P., Brzostek E., Midgley M.G. (2013). The Mycorrhizal-associated Nutrient Economy: A New Framework for Predicting Carbon–Nutrient Couplings in Temperate Forests. New Phytol..

[25] Lewis J.D. (2016). Mycorrhizal Fungi, Evolution and Diversification of. Encyclopedia of Evolutionary Biology.

[26] Rúa M.A., Hoeksema J.D. (2024). Interspecific Selection in a Diverse Mycorrhizal Symbiosis. Sci. Rep..

[27] Põlme S., Bahram M., Jacquemyn H., Kennedy P., Kohout P., Moora M., Oja J., Öpik M., Pecoraro L., Tedersoo L. (2018). Host Preference and Network Properties in Biotrophic Plant-Fungal Associations. New Phytol..

[28] Tedersoo L., Sadam A., Zambrano M., Valencia R., Bahram M. (2010). Low Diversity and High Host Preference of Ectomycorrhizal Fungi in Western Amazonia, a Neotropical Biodiversity Hotspot. ISME J..

[29] Tedersoo L., Jairus T., Horton B.M., Abarenkov K., Suvi T., Saar I., Kõljalg U. (2008). Strong Host Preference of Ectomycorrhizal Fungi in a Tasmanian Wet Sclerophyll Forest as Revealed by DNA Barcoding and Taxon-Specific Primers. New Phytol..

[30] Ding Q., Liang Y., Legendre P., He X., Pei K., Du X., Ma K. (2011). Diversity and Composition of Ectomycorrhizal Community on Seedling Roots: The Role of Host Preference and Soil Origin. Mycorrhiza.

[31] Prieto-Rubio J., Garrido J.L., Alcántara J.M., Azcón-Aguilar C., Rincón A., López-García Á. (2024). Ectomycorrhizal Fungal Network Complexity Determines Soil Multi-Enzymatic Activity. Soil.

[32] Courty P.E., Labbe J., Kohler A., Marc B. (2011). Effect of Poplar Genotypes on Mycorrhizal Infection and Secreted Enzyme Activities in Mycorrhizal and Non-Mycorrhizal Roots. J. Exp. Bot..

[33] Gehring C., Bennett A. (2009). Mycorrhizal Fungal–Plant–Insect Interactions: The Importance of a Community Approach. Environ. Entomol..

[34] Compant S., Van Der Heijden M.G.A., Sessitsch A. (2010). Climate Change Effects on Beneficial Plant-Microorganism Interactions: Climate Change and Beneficial Plant-Microorganism Interactions. FEMS Microbiol. Ecol..

[35] Gehring C.A., Sthultz C.M., Flores-Rentería L., Whipple A.V., Whitham T.G. (2017). Tree Genetics Defines Fungal Partner Communities That May Confer Drought Tolerance. Proc. Natl. Acad. Sci. USA.

[36] Bai X.-N., Hao H., Hu Z.-H., Leng P.-S. (2021). Ectomycorrhizal Inoculation Enhances the Salt Tolerance of Quercus Mongolica Seedlings. Plants.

[37] Chot E., Reddy M.S. (2022). Role of Ectomycorrhizal Symbiosis Behind the Host Plants Ameliorated Tolerance Against Heavy Metal Stress. Front. Microbiol..

[38] Baetz U., Martinoia E. (2014). Root Exudates: The Hidden Part of Plant Defense. Trends Plant Sci..

[39] Garcia K., Delaux P., Cope K.R., Ané J. (2015). Molecular Signals Required for the Establishment and Maintenance of Ectomycorrhizal Symbioses. New Phytol..

[40] Alam B., Lǐ J., Gě Q., Khan M.A., Gōng J., Mehmood S., Yuán Y., Gǒng W. (2021). Endophytic Fungi: From Symbiosis to Secondary Metabolite Communications or Vice Versa? Front. Plant Sci..

[41] Jiarui Y., Haisheng Y. (2023). Research Progress on Symbiotic Interaction and Host Selection Mechanisms of Ectomycorrhizal Fungi. Mycosystema.

[42] Garbaye J. (1994). Tansley Review No. 76 Helper Bacteria: A New Dimension to the Mycorrhizal Symbiosis. New Phytol..

[43] Frey-Klett P., Garbaye J. (2005). Mycorrhiza Helper Bacteria: A Promising Model for the Genomic Analysis of Fungal-Bacterial Interactions: Commentary. New Phytol..

[44] Bonfante P., Anca I.-A. (2009). Plants, Mycorrhizal Fungi, and Bacteria: A Network of Interactions. Annu. Rev. Microbiol..

[45] Venturi V., Keel C. (2016). Signaling in the Rhizosphere. Trends Plant Sci..

[46] Frey-Klett P., Garbaye J., Tarkka M. (2007). The Mycorrhiza Helper Bacteria Revisited. New Phytol..

[47] Martin F. (2016). Molecular Mycorrhizal Symbiosis.

[48] Leveau J.H.J., Preston G.M. (2008). Bacterial Mycophagy: Definition and Diagnosis of a Unique Bacterial–Fungal Interaction. New Phytol..

[49] Boedicker J., Nealson K. (2016). INVITED: Microbial Communication via Quorum Sensing. IEEE Trans. Mol. Biol. Multi-Scale Commun..

[50] Pantigoso H.A., Newberger D., Vivanco J.M. (2022). The Rhizosphere Microbiome: Plant–Microbial Interactions for Resource Acquisition. J. Appl. Microbiol..

[51] Schrey S.D., Schellhammer M., Ecke M., Hampp R., Tarkka M.T. (2005). Mycorrhiza Helper Bacterium Streptomyces AcH 505 Induces Differential Gene Expression in the Ectomycorrhizal Fungus Amanita Muscaria. New Phytol..

[52] Muhammad M.H., Idris A.L., Fan X., Guo Y., Yu Y., Jin X., Qiu J., Guan X., Huang T. (2020). Beyond Risk: Bacterial Biofilms and Their Regulating Approaches. Front. Microbiol..

[53] Roy R., Tiwari M., Donelli G., Tiwari V. (2018). Strategies for Combating Bacterial Biofilms: A Focus on Anti-Biofilm Agents and Their Mechanisms of Action. Virulence.

[54] Röttjers L., Faust K. (2018). From Hairballs to Hypotheses–Biological Insights from Microbial Networks. FEMS Microbiol. Rev..

[55] Santos M.S., Nogueira M.A., Hungria M. (2019). Microbial Inoculants: Reviewing the Past, Discussing the Present and Previewing an Outstanding Future for the Use of Beneficial Bacteria in Agriculture. AMB Expr..

[56] Smith S.E., Read D.J. (2008). Mycorrhizal Symbiosis.

[57] Wang T., Tian Z., Tunlid A., Persson P. (2020). Nitrogen Acquisition from Mineral-associated Proteins by an Ectomycorrhizal Fungus. New Phytol..

[58] Hartmann M., Niklaus P.A., Zimmermann S., Schmutz S., Kremer J., Abarenkov K., Lüscher P., Widmer F., Frey B. (2014). Resistance and Resilience of the Forest Soil Microbiome to Logging-Associated Compaction. ISME J..

[59] Pan L., Cai B. (2023). Phosphate-Solubilizing Bacteria: Advances in Their Physiology, Molecular Mechanisms and Microbial Community Effects. Microorganisms.

[60] Fontaine L., Thiffault N., Paré D., Fortin J.-A., Piché Y. (2016). Phosphate-Solubilizing Bacteria Isolated from Ectomycorrhizal Mycelium of *Picea Glauca* Are Highly Efficient at Fluorapatite Weathering. Botany.

[61] Mavrodi D.V., Ksenzenko V.N., Bonsall R.F., Cook R.J., Boronin A.M., Thomashow L.S. (1998). A Seven-Gene Locus for Synthesis of Phenazine-1-Carboxylic Acid by Pseudomonas Fluorescens 2-79. J. Bacteriol..

[62] Effmert U., Kalderás J., Warnke R., Piechulla B. (2012). Volatile Mediated Interactions Between Bacteria and Fungi in the Soil. J. Chem. Ecol..

[63] Behnsen J., Raffatellu M. (2016). Siderophores: More than Stealing Iron. mBio.

[64] Masri M., Sukmawaty E., Awalia Amir A. (2022). Anti Fungal Activity of Chitinolytic Bacteria Lysinibacillus Fusiformis and Brevibacillus Reuszeri Against the Fungal Pathogens Rhizoctonia Solani and Fusarium Oxysporum. Microbiol. Indones..

[65] Zhang Z., Yuen G.Y., Sarath G., Penheiter A.R. (2001). Chitinases from the Plant Disease Biocontrol Agent, Stenotrophomonas Maltophilia C3. Phytopathology.

[66] Zhu M.-L., Wu X.-Q., Wang Y.-H., Dai Y. (2020). Role of Biofilm Formation by Bacillus Pumilus HR10 in Biocontrol against Pine Seedling Damping-Off Disease Caused by Rhizoctonia Solani. Forests.

[67] Kaur R., Macleod J., Foley W., Nayudu M. (2006). Gluconic Acid: An Antifungal Agent Produced by Pseudomonas Species in Biological Control of Take-All. Phytochemistry.

[68] Olaizola J., Santamaría O., Diez J.J. (2023). In vitro growth of nine edible ectomycorrhizal fungi under a range of pH conditions. Bioagro.

[69] Pereira C.G., Herrera S.J., Machuca H.A., Sánchez O.M. (2007). Effect of pH on the in Vitro Growth of Ectomycorrhizal Fungi Collected from Pinus Radiata Plantations. Bosque.

[70] Soti P.G., Jayachandran K., Koptur S., Volin J.C. (2015). Effect of Soil pH on Growth, Nutrient Uptake, and Mycorrhizal Colonization in Exotic Invasive Lygodium Microphyllum. Plant Ecol..

[71] Zama N., Kirkman K., Mkhize N., Tedder M., Magadlela A. (2022). Soil Acidification in Nutrient-Enriched Soils Reduces the Growth, Nutrient Concentrations, and Nitrogen-Use Efficiencies of Vachellia Sieberiana (DC.) Kyal. & Boatwr Saplings. Plants.

[72] Sehrawat A., Sindhu S.S., Glick B.R. (2022). Hydrogen Cyanide Production by Soil Bacteria: Biological Control of Pests and Promotion of Plant Growth in Sustainable Agriculture. Pedosphere.

[73] Bahar A.K.F., Patandjengi B., Hardiansyah M.Y., Membalik V. (2023). Characterization of Chitinolytic Bacteria Isolated from Ipomea Pes Caprae. IOP Conf. Ser. Earth Environ. Sci..

[74] Miquel Guennoc C., Rose C., Labbé J., Deveau A. (2018). Bacterial Biofilm Formation on the Hyphae of Ectomycorrhizal Fungi: A Widespread Ability under Controls?. FEMS Microbiol. Ecol..

[75] Levy A., Salas Gonzalez I., Mittelviefhaus M., Clingenpeel S., Herrera Paredes S., Miao J., Wang K., Devescovi G., Stillman K., Monteiro F. (2018). Genomic Features of Bacterial Adaptation Toplants. Nat. Genet..

[76] Fernandez C.W., Kennedy P.G. (2016). Revisiting the ‘Gadgil Effect’: Do Interguild Fungal Interactions Control Carbon Cycling in Forest Soils?. New Phytol..

[77] Sterkenburg E., Clemmensen K.E., Ekblad A., Finlay R.D., Lindahl B.D. (2018). Contrasting Effects of Ectomycorrhizal Fungi on Early and Late Stage Decomposition in a Boreal Forest. ISME J..

[78] Averill C., Turner B.L., Finzi A.C. (2014). Mycorrhiza-Mediated Competition between Plants and Decomposers Drives Soil Carbon Storage. Nature.

[79] Yamanaka T. (2003). The Effect of pH on the Growth of Saprotrophic and Ectomycorrhizal Ammonia Fungi in Vitro. Mycologia.

[80] Bending G.D., Read D.J. (1997). Lignin and Soluble Phenolic Degradation by Ectomycorrhizal and Ericoid Mycorrhizal Fungi. Mycol. Res..

[81] Kyaschenko J., Clemmensen K.E., Karltun E., Lindahl B.D. (2017). Below-ground Organic Matter Accumulation along a Boreal Forest Fertility Gradient Relates to Guild Interaction within Fungal Communities. Ecol. Lett..

[82] Lladó S., López-Mondéjar R., Baldrian P. (2017). Forest Soil Bacteria: Diversity, Involvement in Ecosystem Processes, and Response to Global Change. Microbiol. Mol. Biol. Rev..

[83] López-Mondéjar R., Brabcová V., Štursová M., Davidová A., Jansa J., Cajthaml T., Baldrian P. (2018). Decomposer Food Web in a Deciduous Forest Shows High Share of Generalist Microorganisms and Importance of Microbial Biomass Recycling. ISME J..

[84] Kubisch P., Hertel D., Leuschner C. (2016). Fine Root Productivity and Turnover of Ectomycorrhizal and Arbuscular Mycorrhizal Tree Species in a Temperate Broad-Leaved Mixed Forest. Front. Plant Sci..

[85] Teste F.P., Jones M.D., Dickie I.A. (2020). Dual-mycorrhizal Plants: Their Ecology and Relevance. New Phytol..

[86] Mahmoudi N., Mahdhi M., Abddaiem R., Bessadok K., Mars M. (2019). Arbuscular Mycorrhizal Colonization of Selected Herbaceous Plants under Arid Protected Area in Tunisia. Soil. Sci. Plant Nutr..

[87] Torrecillas E., Alguacil M.M., Roldán A. (2012). Host Preferences of Arbuscular Mycorrhizal Fungi Colonizing Annual Herbaceous Plant Species in Semiarid Mediterranean Prairies. Appl. Environ. Microbiol..

[88] Kadowaki K., Yamamoto S., Sato H., Tanabe A.S., Hidaka A., Toju H. (2018). Mycorrhizal Fungi Mediate the Direction and Strength of Plant–Soil Feedbacks Differently between Arbuscular Mycorrhizal and Ectomycorrhizal Communities. Commun. Biol..

[89] Ferlian O., Cesarz S., Craven D., Hines J., Barry K.E., Bruelheide H., Buscot F., Haider S., Heklau H., Herrmann S. (2018). Mycorrhiza in Tree Diversity–Ecosystem Function Relationships: Conceptual Framework and Experimental Implementation. Ecosphere.

[90] Awad A., Majcherczyk A., Schall P., Schröter K., Schöning I., Schrumpf M., Ehbrecht M., Boch S., Kahl T., Bauhus J. (2019). Ectomycorrhizal and Saprotrophic Soil Fungal Biomass Are Driven by Different Factors and Vary among Broadleaf and Coniferous Temperate Forests. Soil. Biol. Biochem..

[91] Tedersoo L., Bahram M. (2019). Mycorrhizal Types Differ in Ecophysiology and Alter Plant Nutrition and Soil Processes. Biol. Rev..

[92] Baptista P., Guedes De Pinho P., Moreira N., Malheiro R., Reis F., Padrão J., Tavares R., Lino-Neto T. (2021). In Vitro Interactions between the Ectomycorrhizal Pisolithus Tinctorius and the Saprotroph Hypholoma Fasciculare Fungi: Morphological Aspects and Volatile Production. Mycology.

[93] Rousk J., Bååth E. (2011). Growth of Saprotrophic Fungi and Bacteria in Soil: Growth of Saprotrophic Fungi and Bacteria in Soil. FEMS Microbiol. Ecol..

[94] Olivain C., Humbert C., Nahalkova J., Fatehi J., L’Haridon F., Alabouvette C. (2006). Colonization of Tomato Root by Pathogenic and Nonpathogenic Fusarium Oxysporum Strains Inoculated Together and Separately into the Soil. Appl. Environ. Microbiol..

[95] Elhamouly N.A., Hewedy O.A., Zaitoon A., Miraples A., Elshorbagy O.T., Hussien S., El-Tahan A., Peng D. (2022). The Hidden Power of Secondary Metabolites in Plant-Fungi Interactions and Sustainable Phytoremediation. Front. Plant Sci..

[96] Raudaskoski M., Kothe E. (2015). Novel Findings on the Role of Signal Exchange in Arbuscular and Ectomycorrhizal Symbioses. Mycorrhiza.

[97] Peng Y., Li S.J., Yan J., Tang Y., Cheng J.P., Gao A.J., Yao X., Ruan J.J., Xu B.L. (2021). Research Progress on Phytopathogenic Fungi and Their Role as Biocontrol Agents. Front. Microbiol..

[98] Fernández N., Knoblochová T., Kohout P., Janoušková M., Cajthaml T., Frouz J., Rydlová J. (2022). Asymmetric Interaction Between Two Mycorrhizal Fungal Guilds and Consequences for the Establishment of Their Host Plants. Front. Plant Sci..

[99] Peay K.G. (2016). The Mutualistic Niche: Mycorrhizal Symbiosis and Community Dynamics. Annu. Rev. Ecol. Evol. Syst..

